# Editorial: Gravitational Physiology, Aging and Medicine

**DOI:** 10.3389/fphys.2019.01338

**Published:** 2019-10-25

**Authors:** Nandu Goswami, Jack J. W. A. van Loon, Andreas Roessler, Andrew P. Blaber, Olivier White

**Affiliations:** ^1^Division of Physiology, Otto Loewi Research Center for Vascular Biology, Immunology and Inflammation, Medical University of Graz, Graz, Austria; ^2^Department of Health Sciences, Alma Mater Europaea Maribor, Maribor, Slovenia; ^3^Department Oral and Maxillofacial Surgery/Pathology, Amsterdam UMC, Vrije Universiteit Medical Center, Academic Centre for Dentistry Amsterdam (ACTA), Amsterdam, Netherlands; ^4^Department of Biomedical Physiology and Kinesiology, Simon Fraser University, Burnaby, BC, Canada; ^5^INSERM UMR1093-CAPS, Université Bourgogne Franche-Comté, UFR des Sciences du Sport, Dijon, France

**Keywords:** spaceflight, aging, bedrest, falls, deconditioning, immersion, countermeasures

Physiological deconditioning changes induced by spaceflight are similar to those that occur in aging, thus leading to, for example, a greater incidence of syncope and falls and to a decrease in quality of life. The Research Topic “*Gravitational Physiology, Aging and Medicine*” examines effects of gravitational changes on human physiology, with applications to geriatrics and clinical medicine. The Research Topic also aimed at promoting national and international networks that include activities used to stimulate student-oriented learning.

The presence of gravity is known to influence human physiology and behavior. For instance, gravitational loads are required to maintain a healthy musculoskeletal system. Indeed, upon standing, ~500 mL of blood move into the lower limbs within seconds. This can compromise venous return, and hence blood pressure, leading to falls. Gravity also influences the way we interact with the environment because it provides a strong reference to align multimodal information such as vision and proprioception, critical for balance. Fortunately, the body, through neural mechanisms, compensates rapidly to postural changes, acting to maintain mean arterial pressure and prevent orthostatic intolerance.

The microgravity environment of spaceflight causes cardiovascular, neurovestibular and musculoskeletal changes, including bone loss and muscle atrophy. The observed physiological changes lead to deconditioning and impaired responses to gravitational stress on return to Earth. These changes share important common features with the deconditioning and impaired functions due to the aging process. Such changes that are seen in astronauts as well as in older persons lead to greater incidence of orthostatic intolerance. In elderly persons, this can lead to falls which are associated with head injuries and/or bone fractures. Such injuries and fractures are often associated with long-term immobilization and, consequently, further deconditioning, a vicious cycle from which patients may not be able to recover. Several papers in this Research Topic specifically address the parallel mechanisms between spaceflight deconditioning and aging (Goswami; Goswami et al.; Strollo et al.; Siamwala et al.).

Furthermore, studies aimed at understanding spaceflight-induced deconditioning often use ground-based analogs such as bedrest confinement and wet and dry water immersion (Tomilovskaya et al.). Such studies provide unique insights into the role of short and long term bedrest as well as immersion on physiological responses (discussed in Tomilovskaya et al.; de Abreu et al.; Kermorgant et al.; Šarabon et al.; Stavrou et al.; Gennser et al.). The knowledge gained from those investigations is important for developing countermeasures against deconditioning induced by spaceflight and aging. Such countermeasures are discussed in Maggioni et al., Temple et al., White et al., and Marusic et al..

Bed confinement is a paramount problem in older persons. Hence, data from bedrest studies are useful for understanding the deconditioning effects of this kind of immobilization in this population, which is a rapidly growing segment of the overall population. Bedrest can lead to postural control deficiencies and orthostatic hypotension which are major contributors to falls in the elderly (see Goswami). Naturally, it can be expected that integrating information inherited from space environments and ground-based models of deconditioning will provide novel perspectives and innovative approaches for expanding knowledge in both space physiology and aging medicine. For example, scientific insights and methodologies developed in space science research of orthostatic intolerance can be exploited to study cardiovascular, cerebrovascular, and postural sensory motor control systems in males and females (reviewed in Evans et al.). This Research Topic is a perfect example of translational and multidisciplinary research.

## Topics Covered

This Research Topic received 45 papers for peer-review. Following rigorous reviewing of abstracts and full papers, and after careful screening of revised manuscripts, 36 were selected for publication. The areas covered include aspects related to physiology of gravitational (un-)loading, effects of bedrest confinement, dry immersion, as well as aging-related physiological deconditioning. In addition, this Research Topic has examined the ways in which knowledge of life in space—and the countermeasures developed in space to overcome spaceflight-induced effects—can help life on Earth. Like the possible application of large radius artificial gravity for research, possible treatment and training (van Loon et al., 2012). While most of the contributions were based on studies of responses in human participants, some used animal models to investigate more fundamental mechanisms (Gambara et al.; Giuliani et al.; Ling et al.). This Research Topic included both original research articles and reviews. The collection of articles, which also examine sex-based diiferences in physiological responses (Masatli et al.; White et al., 2019; Evans et al.), can be broadly classified as:

1. Overaching reviews and original articles related to **acute and chronic effects of spaceflight**. These papers included those that examined stress-related shift toward inflammaging in long-duration spaceflight (Buchheim et al.), effects of hypogravity on human locomotion (Lacquaniti et al.), including arm kinematics and postural strategy in whole-body reaching movements that occur in microgravity (Macaluso et al.), and venous function changes during long duration spaceflights (Fortrat et al.). Spinal health during loading and unloading that occurs in spaceflight (Green and Scott) as well as biomechanical and cardiopulmonary responses to partial gravity (Richter et al.) are also addressed.
2. Effects of **ground-based models of microgravity** such as **bedrest** confinement (Šarabon et al.; Stavrou et al.) and **dry immersion** (Tomilovskaya et al.; de Abreu et al.; Kermorgant et al.) on physiological responses are discussed.
3. Data from **parabolic flights** on how sensorimotor reorganization of arm kinematics and postural strategy for functional whole-body reaching movements occur in microgravity was investigated in detail (Macaluso et al.).
4. The impact of **artificial gravity on motor and physiological functions** (Barbiero et al.; White et al.; Winter et al.) and the potential role of artificial gravity **in prevention of microgravity-/bedrest induced physiological deconditioning** (Masatli et al.; Evans et al.) are present in our topic.
5. Physiology of **spaceflight-induced deconditioning** and its **similiarties with aging** (Strollo et al.; Goswami; Goswami et al.; Siamwala et al.).
6. **Countermeasures that could alleviate effects of spaceflight, bedrest and/or aging**. These include exercise (Maggioni et al.), stochastic resonance (Temple et al.; White et al.), and cognitive training (Marusic et al.).
7. Original work and **general** reviews related to **innovative measurements techniques and advances in gravitational physiology and medicine**. These include effects of orthosatic loading on coagulaton in patients (Cvirn et al.), limb temperature as indicator of orthostatic intolerance (Opatz et al.), aortic compliance and cerebral blood flow during orthostatic loading in endurance athletes (Tomoto et al.), and lower body negative pressure (LBNP) (Goswami et al., 2008, 2019) induced non-linear heart rate and blood pressure interactions (Verma et al.). Finally, the underlying mechanisms that contribute to muscle atrophy during mechanical unloading (Gao et al.) as well as how alterations in leg muscle-tendon unit biomechanical properties occur with aging and mechanical loading (McCrum et al.) are also presented.

## Author Contributions

NG, JL, AR, AB, and OW were responsible for this Research Topic design, invitation to potential authors, reviews of abstracts, and submitted manuscripts and finally, in preparation of the editorial.

### Conflict of Interest

The authors declare that the research was conducted in the absence of any commercial or financial relationships that could be construed as a potential conflict of interest.
